# *Astragalus* polysaccharides decrease proliferation, migration, and invasion but increase apoptosis of human osteosarcoma cells by up-regulation of microRNA-133a

**DOI:** 10.1590/1414-431X20187665

**Published:** 2018-11-14

**Authors:** Yanchen Chu, Yuan Fang, Jingwei Chi, Jing Li, Dongyang Zhang, Yunwen Zou, Zhijie Wang

**Affiliations:** 1 Department of Spinal Surgery, The Affiliated Hospital of Qingdao University, Qingdao, Shandong, China; 2 Department of Joint Surgery, The Affiliated Hospital of Qingdao University, Qingdao, Shandong, China; 3 Key Laboratory of Thyroidism, The Affiliated Hospital of Qingdao University, Qingdao, Shandong, China; 4 Department of Gynaecology and Obstetrics, The Affiliated Hospital of Qingdao University, Qingdao, Shandong, China; 5 Department of Orthopedics, Laixi People's Hospital, Laixi, Shandong, China

**Keywords:** Osteosarcoma, *Astragalus* polysaccharides, Anti-tumor, microRNA-133a, JNK

## Abstract

Osteosarcoma (OS) has a high incidence, malignity, and frequency of recurrence and metastasis. In this study, we aimed to explore the potential anti-cancer effects of *Astragalus* polysaccharides (APS) on human OS MG63 cells as well as underlying mechanisms. Viability of MG63 cells was assessed by CCK-8 assay to determine the adequate concentration of APS. Then, effects of APS on MG63 cell proliferation, cell cycle distribution, apoptosis, and migration and invasion were analyzed by BrdU incorporation, PI staining, flow cytometry, and transwell assays, respectively. The expression levels of proteins involved in these physiological processes were assessed by western blot analysis. Afterwards, miR-133a level in APS-treated cells was determined by qRT-PCR, and whether APS affected MG63 cells through regulation of miR-133a was determined. Finally, the activation of c-Jun N-terminal protein kinase (JNK) pathway was detected. We found that APS treatment suppressed the viability, proliferation, migration, and invasion of MG63 cells, as well as induced cell apoptosis. Moreover, APS enhanced the expression of miR-133a in MG63 cells. Knockdown of miR-133a reversed the APS treatment-induced MG63 cell proliferation, migration and invasion inhibition, as well as cell apoptosis. Furthermore, APS inactivated JNK pathway in MG63 cells. Knockdown of miR-133a reversed the APS treatment-induced inactivation of JNK pathway in MG63 cells. To conclude, APS repressed proliferation, migration, and invasion while induced apoptosis of OS MG63 cells by up-regulating miR-133a and then inactivating JNK pathway.

## Introduction

As the most common aggressive cancer in the human skeletal system, osteosarcoma (OS) is becoming the second leading cause of cancer-related deaths in children and adolescents (1,2). Tumor metastasis is the main reason for the death of patients with OS (3). Before diagnosis, about 15–20% of OS patients present metastasis, and 40% of patients will develop metastasis during treatments (4,5). Currently, with the development of surgical removal and multiple-targets therapy, the prognosis of OS has been improved significantly (6). However, 30% of localized OS and 70% of metastatic OS still have a poor prognosis (7). Therefore, more effective and suitable therapeutic agents should be identified to further improve the survival of OS.

*Astragalus* polysaccharides (APS) are the main active ingredients isolated from the root of *Astragalus membranaceus* (Fisch.) Bunge with diverse bio-activities. For example, Chen et al. (8) showed that APS could protect myocardium in diabetic hamsters by improving myocardial glycolipid metabolic disorder. Liu et al. (9) indicated that APS could protect liver from ionizing radiation-induced injury by reducing oxidative stress in animals. The study from Guo et al. (10) reported that APS could be used as a potential anti-Epstein-Barr virus drug. The anti-inflammatory effects of APS have been reported both *in vivo* and *in vitro* (11,12). Recently, the anti-cancer activity of APS has been identified, which demonstrated that APS could inhibit liver cancer in murine H22 hepatocarcinoma model (13). In human hepatocellular carcinoma cells, APS has been found to significantly reduce cell viability and induce apoptosis (14). However, the role of APS in OS remains unclear.

Although the anti-cancer effects of APS have been reported, studies on the underlying mechanisms are limited. MicroRNAs (miRNAs/miRs) are short, non-coding RNAs in eukaryotic cells that play key roles in the regulation of protein synthesis thereby participating in multiple biological processes (15). Numerous miRNAs have been identified to be involved in the progression of OS, acting as oncogenes or tumor suppressors. For example, miR-130b has been found to promote proliferation and inhibit apoptosis of OS cells through regulating the Wnt pathway (16). Conversely, miR-26a has been reported to repress the stem cell-like phenotype and tumor growth of OS cells by targeting Jagged1 (17). Moreover, a previous study reported that APS down-regulated miR-721 and thereby exerted insulin resistance in 3T3-L1 adipocytes (18). Therefore, we hypothesized that APS might affect OS cells through regulation of miRNAs.

In our study, we explored the functional roles of APS in proliferation, apoptosis, migration, and invasion of OS cells. Moreover, the underlying molecular mechanism associated with miRNAs and JNK signaling pathway was investigated.

## Material and Methods

### Cell culture and treatment

Human OS cell line MG63 was obtained from the Institute of Biochemistry and Cell Biology, Chinese Academy of Sciences (China). MG63 cells were maintained in high glucose Dulbecco's modified Eagle's medium (DMEM; Invitrogen, USA) containing 10% (v/v) fetal bovine serum (Invitrogen) and 1% (v/v) penicillin-streptomycin (100X, Gibco, Life Technologies, USA) at 37°C with 5% CO_2_.

APS were obtained from Boster Biology Corporation (China) and dissolved in pure water following the manufacturer's instruction. For APS treatment, MG63 cells were incubated in DMEM containing 0–20 mg/mL APS at 37°C for 24 h.

### Cell viability assay

Viability of MG63 cells after APS treatment was determined by Cell Counting Kit-8 (CCK-8) assay. Briefly, cells were seeded into 96-well plates with a density of 5×10^3^ cells per well. After incubation at 37°C overnight, the culture medium was replaced by DMEM containing 0-20 mg/mL APS. After stimulation for 24 h, 10 μL of CCK-8 solution (Dojindo Molecular Technologies, USA) was added to each well, and the plate was maintained at 37°C for 1 h Subsequently, the absorbance of each well at 450 nm was measured using a Microplate Reader (Bio-Rad, USA). Cell viability (%) was calculated by average absorbance of APS treatment group/average absorbance of control group × 100%.

### Cell transfection

MiR-133a inhibitor and its negative control (NC) were synthesized by GenePharma Co. (China). The sequence of miR-133a inhibitor was 5′-CAGCUGGUUGAAGGGGACCAAA-3′. The sequence of NC was 5′-UCACAACCUCCUAGAAAGAGUAGA-3′. For transient transfection, 100 nM miRNAs were transfected into MG63 cells using Lipofectamine 3000 reagent (Invitrogen) following the manufacturer's instructions.

### Cell proliferation assay

Proliferation of MG63 cells was analyzed by 5-bromo-2′-deoxyuridine (BrdU) incorporation assay using a Cell Proliferation ELISA kit (Roche, Germany). Briefly, cells were plated in 96-well plates and maintained overnight. Then, after relevant treatments, cells were incubated in BrdU labeling solution for 3 h at 37°C, and subjected to recommended protocols from the manufacturer. Finally, the absorbance of each well at 450/550 nm was measured by a Microplate Reader (Bio-Rad).

### Cell cycle analysis

MG63 cell cycle distribution after APS treatment was analyzed using PI staining and flow cytometer. Briefly, MG63 cells were seeded into 24-well plates with a density of 3×10^4^ per well. After incubation at 37°C overnight, the culture medium was replaced by DMEM containing 10 mg/mL APS for 24 h. Then, cells in each group were harvested, washed twice with cold phosphate buffered saline (PBS), fixed with 70% ice-cold ethanol at –20°C overnight, washed twice with cold PBS, incubated with 100 mg/mL RNase A for 30 min at 37°C, stained with 50 mg/mL PI in the dark for 30 min, and subjected to flow cytometry analysis. For each experiment, 5×10^3^ cells were recorded. The obtained results were analyzed by Cell Quest software (Beckton Dickinson Immunocytometry Systems, USA).

### Cell apoptosis assay

Apoptosis of MG63 cells was assessed by double staining using a FITC Annexin V/Dead Cell Apoptosis Kit (Invitrogen). Briefly, after corresponding treatments, cells were collected, washed with PBS, and re-suspended in binding buffer. Then, 5 μL FITC-Annexin V and 5 μL PI were added to the binding buffer, followed by incubation at room temperature in the dark for 15 min. At least 2×10^4^ cells were acquired using a flow cytometer (BD Bioscience, USA). Percentage of apoptotic cells was analyzed by FlowJo software (Tree Star, USA).

### Migration and invasion assays

Migration of MG63 cells was determined by Transwell assay using the modified 24-well Boyden chambers (8-μm pore size, Corning Incorporated, USA). Cell invasion was assessed similarly with the cell migration assay, except that the transwell membranes were pre-coated with Matrigel (BD Biosciences). In brief, after relevant treatments, MG63 cells were re-suspended in 200 μL FBS-free medium and seeded into the upper compartment. The lower compartment was filled with 600 μL complete medium. The chambers were transferred to a humidified incubator at 37°C for 48 h. After that, the cells on the upper surface of the inserts were removed carefully with a cotton swab, and the cells on the lower side of the filter were fixed in 100% methanol. Then, the fixed cells were stained with crystal violet solution and cells in five randomly chosen fields were counted microscopically.

### Quantitative reverse transcription PCR (qRT-PCR)

Total RNAs in MG63 cells were isolated using a PureLink RNA Mini Kit (Thermo Fisher Scientific, USA). Then, RNA was quantified using a NanoDrop 1000 Spectrophotometer (Thermo Fisher Scientific) and reverse-transcribed using the Universal cDNA Synthesis Kit II (Takara, China), following the manufacturer's protocol. After that, real-time PCR was performed using the ExiLENT SYBR® Green master mix (Takara) according to the supplier's protocol. The PCR conditions were as follows: 95°C for 10 min, 40 cycles of 95°C for 10 s, and 60°C for 1 min. Fold-change of miR-133a was calculated by the 2^−ΔΔCt^ method (19), and U6 expression was used to normalize the results.

### Western blot analysis

Total proteins in MG63 cells were prepared in radioimmunoprecipitation (RIPA) buffer supplemented with protease inhibitor (Sigma, USA). Total proteins in cytoplasm of MG63 cells were isolated using Cytoplasmic Protein Extraction kit (Boster Biology Corporation). After centrifugation at 12,000 *g* at 4°C for 20 min, protein content in the supernatants was determined by a bicinchoninic acid (BCA) assay kit (Thermo Scientific). Then, 40 μg protein samples were separated by SDS-PAGE and transferred to nitrocellulose membranes, followed by blocking in fat-free milk. The membranes were incubated with primary antibodies against cyclinD1 (ab134175), p21 (ab109199), cytochrome C (ab90529), B cell lymphoma-2 (Bcl-2, ab196495), Bcl-2-associated X protein (Bax, ab182733), cleaved caspase-3 (ab2302), cleaved caspase-9 (ab2324), matrix metalloproteinase (MMP)-2 (ab97779), MMP-9 (ab137867), vimentin (ab137321), c-Jun (ab32137), phospho (p)-c-Jun (ab32385), β-actin (ab8229, all Abcam, Cambridge, UK), c-Jun N-terminal protein kinase (JNK; 9252), or p-JNK (9251, both Cell Signaling Technology, USA) at 4°C overnight. Membranes were then incubated with secondary antibody conjugated to HRP (goat anti-rabbit, ab205718, Abcam) at room temperature for 1 h. Proteins in the membranes were detected using an enhanced Pierce chemiluminescence kit (Sigma), and the optical density of the bands was determined by ImageJ 1.47 software (National Institutes of Health, USA).

### Statistical analysis

Data are reported as means±SE of three independent experiments. Statistical analysis was performed using GraphPad Prism 5 software (GraphPad, USA). Two-tailed Student's *t*-test was used for comparisons between two groups, and one-way analysis of variance (ANOVA) was used for comparisons among three groups. P<0.05 was considered to be a significant difference.

## Results

### APS repressed proliferation and induced apoptosis of MG63 cells

**Figure 1.**
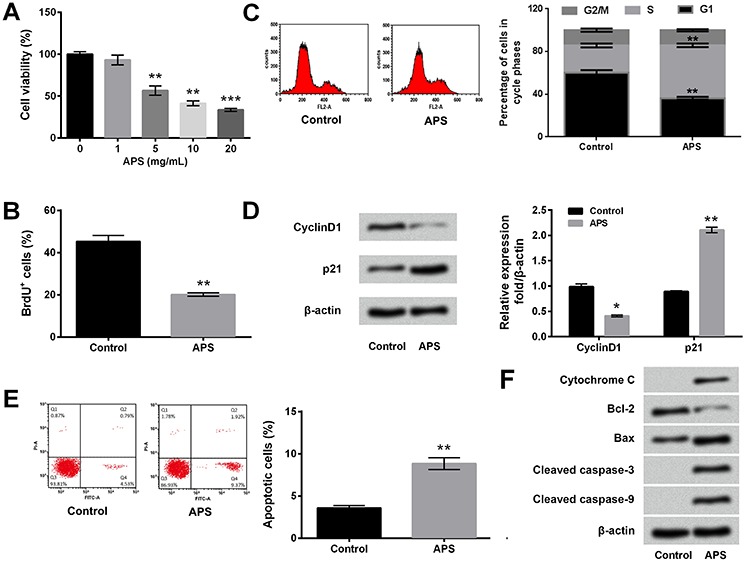
*Astragalus* polysaccharides (APS) reduced proliferation and induced apoptosis of MG63 cells. MG63 cells were treated by 1-20 mg/mL APS. Non-treated cells acted as control. *A*, Cell viability was detected by CCK-8 assay. *B*, Percentage of BrdU positive cells was measured by BrdU incorporation assay. *C*, Cell cycle distribution was assessed using PI staining and flow cytometry. *D*, Expression levels of proliferation-associated proteins were evaluated by western blot analysis. *E*, Percentage of apoptotic cells was determined by FITC Annexin V/Dead Cell Apoptosis kit and flow cytometry. *F*, Expression levels of apoptosis-associated proteins were evaluated by western blot analysis. Data are reported as means±SE of three independent experiments. *P<0.05; **P<0.01; ***P<0.001 (ANOVA or *t*-test).

Viability of MG63 cells after 0–20 mg/mL APS treatment was measured to determine the adequate concentration of APS. Cell viability was significantly reduced by 5-20 mg/mL APS treatment compared to non-treated cells (P<0.01 or P<0.001) (Figure 1A). The APS concentration of 10 mg/mL was chosen for subsequent experiments. Then, effects of APS on MG63 cell proliferation, cell cycle distribution, and apoptosis were explored. Percentage of BrdU positive cells in the APS treatment group was lower than that in the control group (P<0.01, Figure 1B). The results of Figure 1C show that APS treatment significantly induced MG63 cell cycle S phase arrest (P<0.01). Meanwhile, the expression of CyclinD1 expression was down-regulated (P<0.05) and the expression of p21 was up-regulated (P<0.01) after 10 mg/mL APS treatment (Figure 1D). As seen in Figure 1E, the percentage of apoptotic cells in the APS group was dramatically higher than that in the control group (P<0.01). Accordingly, APS stimulation induced down-regulation of Bcl-2 and up-regulations of Bax, cleaved caspase-3, and cleaved caspase-9 in MG63 cells (Figure 1E). The expression of cytochrome C in cytoplasmic of MG63 cells was also increased after APS treatment. These results indicated that APS repressed proliferation and induced apoptosis of MG63 cells.

### APS suppressed migration and invasion of MG63 cells

**Figure 2.**
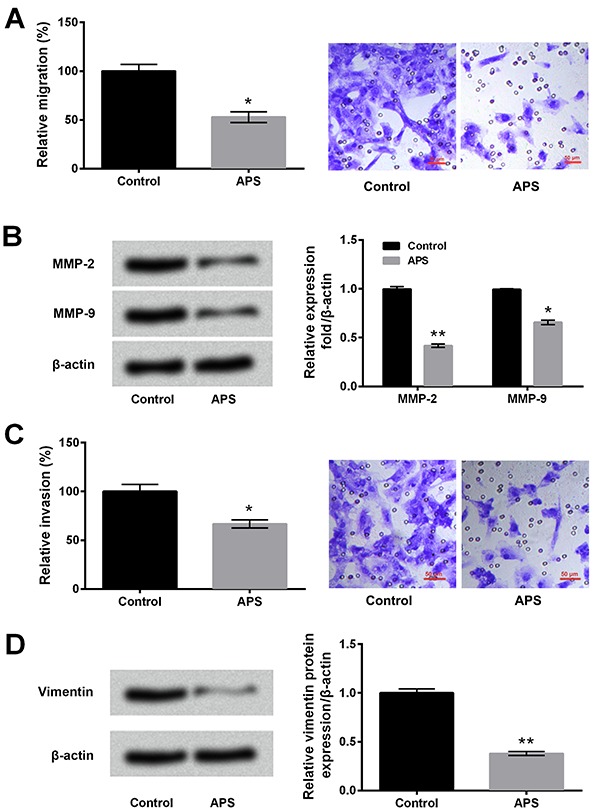
*Astragalus* polysaccharides (APS) suppressed migration and invasion of MG63 cells. MG63 cells were treated with 10 mg/mL APS. Non-treated cells acted as control. *A*, Cell migration was measured with the transwell migration assay. *B*, Expression levels of migration-associated proteins were assessed by western blot analysis. *C*, Cell invasion was detected with the transwell invasion assay. *D,* Expression of invasion-associated proteins were evaluated by western blot analysis. MMP: matrix metalloproteinase. Data are reported as means±SE of three independent experiments. *P<0.05; **P<0.01 compared to control (*t-test*).

Compared with the control group, the relative migration of MG63 cells was significantly decreased after 10 mg/mL APS treatment (P<0.05, Figure 2A). The expression levels of MMP-2 and MMP-9 in MG63 cells were down-regulated after APS stimulation (P<0.05 or P<0.01, Figure 2B). Similarly, APS reduced invasion of MG63 cells (P<0.05, Figure 2C) and down-regulated the expression of Vimentin (P<0.01, Figure 2D) in MG63 cells. These results suggested that APS inhibited migration and invasion of MG63 cells.

### APS induced up-regulation of miR-133a in MG63 cells

**Figure 3.**
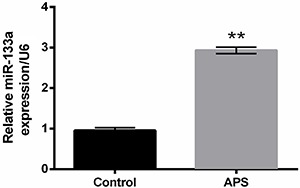
*Astragalus* polysaccharides (APS) induced up-regulation of miR-133a in MG63 cells. MG63 cells were stimulated with 10 mg/mL APS. Non-treated cells acted as control. Expression of miR-133a was determined by quantitative reverse transcription PCR. Data are reported as means±SE of three independent experiments. **P*<*0.01 (*t*-test).

As presented in Figure 3, the level of miR-133a in the APS treatment group was higher than that in the control group (P<0.01), implying that miR-133a might participate in the anti-cancer effects of APS on MG63 cells.

### APS exerted anti-cancer effects on MG63 cells by up-regulating miR-133a

**Figure 4.**
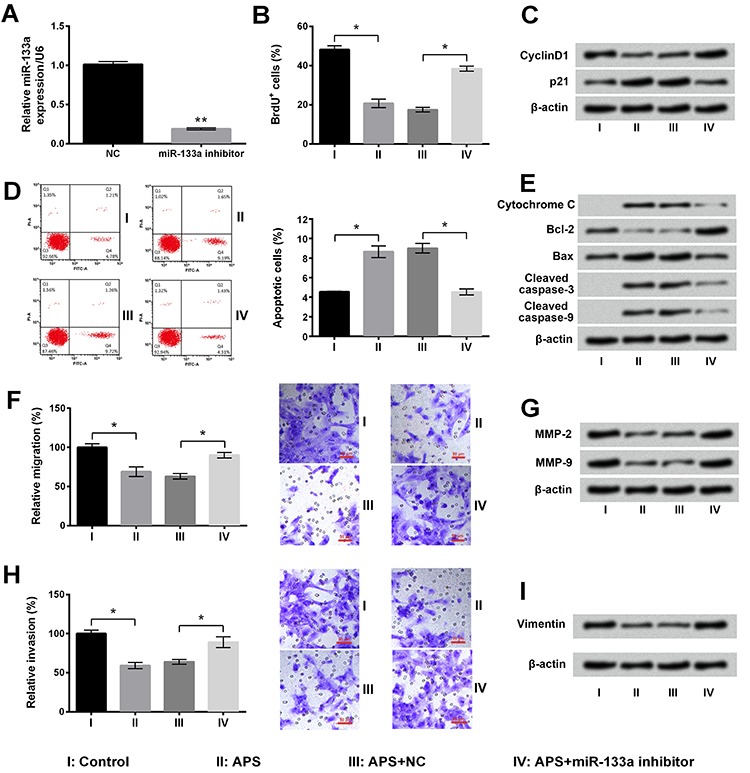
Effects of *Astragalus* polysaccharides (APS) on MG63 cells were reversed by miR-133a inhibition. *A*, After miR-133a inhibitor transfection, the expression of miR-133a in MG63 cells was determined by quantitative reverse transcription PCR. Transfected and non-transfected cells were stimulated with 10 mg/mL APS. Non-treated cells acted as control (NC). *B*, Percentage of BrdU positive cells was detected by BrdU incorporation assay. *C*, Expression levels of proliferation-associated proteins were assessed by western blot analysis. *D*, Percentage of apoptotic cells was measured by flow cytometry. *E*, Expression levels of apoptosis-associated proteins were evaluated by western blot analysis. *F*, Cell migration was determined by transwell migration assay. *G*, Expression levels of migration-associated proteins were detected by western blot analysis. MMP: matrix metalloproteinase. *H*, Cell invasion was measured by transwell invasion assay. *I*, Expression of invasion-associated protein was evaluated by western blot analysis. Data are reported as means±SE of three independent experiments. *P<0.05; **P<0.01 (ANOVA).

The results in Figure 4A show that miR-133a was significantly reduced after miR-133a inhibitor transfection compared with the NC group (P<0.01). APS treatment-induced MG63 cell proliferation, migration, and invasion inhibition, cell apoptosis, and expression of proteins associated with these physiological processes were all significantly reversed by miR-133a knockdown (P<0.05 or P<0.01) (Figure 4B-4I). Taken together, these finding suggested that APS exerted anti-cancer effects on MG63 cells might via up-regulation of miR-133a.

### APS inhibited JNK pathway in MG63 cells by up-regulating miR-133a

**Figure 5.**
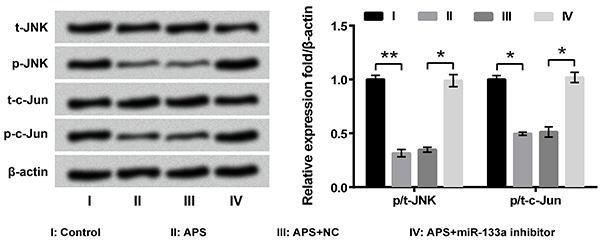
*Astragalus* polysaccharides (APS) inhibited JNK pathway through up-regulating miR-133a in MG63 cells. Transfected and non-transfected MG63 cells were stimulated with 10 mg/mL APS. Non-treated cells acted as control. Expression levels of proteins involved in the JNK pathway were assessed by western blot analysis. Data are reported as means±SE of three independent experiments. *P<0.05; **P<0.01 (ANOVA).

Finally, the activation of JNK pathway in MG63 cells after APS treatment and/or miR-133a transfection was evaluated using western blotting. The results showed that the phosphorylated levels of JNK and c-Jun were both decreased after APS treatment (P<0.05 or P<0.01, Figure 5). Furthermore, the APS-induced phosphorylated levels reduction of p-JNK and p-c-Jun were reversed by miR-133a inhibitor transfection (both P<0.05). These results illustrated that APS inactivated the JNK pathway through up-regulating miR-133a in MG63 cells.

## Discussion

Tumor cells are able to thrive through promoted proliferation and inhibited apoptosis (20). Thus, inhibition of proliferation and inducement of apoptosis are considered two important therapeutic methods for tumor treatment, including OS treatment. BrdU is a thymidine analog that can be incorporated into DNA during replication; therefore, BrdU is widely used for labeling proliferating cells (21). Herein, the lower percentage of BrdU-positive cells after APS stimulation illustrated that APS could repress MG63 cell proliferation. CyclinD1 is a key mitogen sensor that promotes the conversion from the G1 phase to the S phase and thereby promotes mitosis (22). p21 is the cyclin-dependent kinase inhibitor, which could regulate the G-phase checkpoint, induce cell cycle arrest, and impair DNA replication (23). In our study, the APS-induced down-regulation of cyclinD1 and up-regulation of p21 consolidated the anti-proliferative role of APS in MG63 cells. Flow cytometry results illustrated the pro-apoptotic role of APS. Moreover, APS down-regulated the anti-apoptotic Bcl-2 and up-regulated the pro-apoptotic Bax, leading to activation of caspase-9 and caspase-3. The expression of cytochrome in cytoplasm of MG63 cells was also increased after APS treatment. These alterations of apoptosis-associated proteins indicate that APS could induce MG63 cell apoptosis through mitochondrial and caspase-dependent pathways.

OS is a tumor with metastatic potential and metastasis is considered the leading cause of poor outcomes in OS patients (24,25). Since migration and invasion are two major characteristics of metastasis, influence of APS on migration and invasion of MG63 cells was further explored. The extracellular matrix (ECM) is a complex network that occupies the space between cells and maintains tissue integrity (26). Excessive degradation of ECM is a crucial process, by which cancer cells begin to migrate and invade (27). MMP-2 and MMP-9 are important molecules that degrade ECM and basement membrane (28). Vimentin, an intermediate filament protein, is widely accepted to be a metastasis marker and plays essential roles in the formation of invadopodia (29,30). In our study, the down-regulations of MMP-2, MMP-9, and Vimentin reflected the inhibitory effects of APS on migration and invasion of MG63 cells, suggesting that APS exerted anti-cancer role in OS also by inhibiting cell migration and invasion.

MiR-133a has been reported as a tumor suppressor in several cancer types, such as glioma, colorectal cancer, etc. (31,32). For OS, miR-133a down-regulation is associated to unfavorable prognosis in patients with OS (33). OS cell proliferation, invasion, and metastasis were all repressed by miR-133a (34). Another study also stated that miR-133a inhibited proliferation and induced apoptosis of OS cells (35). Thus, we supposed that there might be an interaction between miR-133a expression and APS stimulation. We found that miR-133a expression was up-regulated in APS treatment group. More importantly, miR-133a knockdown reversed the effects of APS on MG63 cell proliferation, apoptosis, migration, and invasion, as well as expression levels of associated proteins. These results indicate that APS possess anti-tumorous activity through up-regulating miR-133a in MG63 cells.

Fromigué et al. (36) reported the regulatory role of JNK in MMP-2 activity and invasion process in OS cells. c-Jun is a crucial mediator and it can be phosphorylated by JNK, followed by activation of MMP-2 promoter (37). Consequently, the phosphorylation of JNK and c-Jun was studied. Some miRs, including miR-223 (38), miR-127 (39), and miR-373 (40), have been found to be involved in the regulation of JNK pathway in OS cells. Herein, we proposed that miR-133a might also participate in the regulation of JNK pathway in MG63 cells. Thus, we also analyzed the activation of JNK pathway in MG63 cells after APS treatment and/or miR-133a inhibitor transfection. We found that JNK pathway was inhibited by APS, which might be a rational explanation for the down-regulation of MMPs and inhibition of metastasis. We also found that APS-induced inactivation of JNK pathway was reversed by miR-133a knockdown in MG63 cells. These findings implied that APS exerted anti-cancer roles in MG63 cells by up-regulating miR-133a and then inactivating JNK pathway.

To summarize, we reported for the first time that APS exerted anti-tumorous activity in OS cells by inhibiting proliferation, migration, and invasion, and inducing apoptosis. In addition, we pointed out that miR-133a and JNK pathway were involved in the anti-cancer effects of APS on OS cells. Our study provides a theoretical basis for further exploring the treatment of OS using APS. More *in vitro* and *in vivo* studies are still needed.
